# Surgical strategy in a patient with combined infective endocarditis of pseudomonas putida and metisiline sensitive staphylococcus, complicated with splenic infarction cerebral abscess.

**DOI:** 10.1186/1749-8090-10-S1-A220

**Published:** 2015-12-16

**Authors:** Ali Gürbüz, Ufuk Yetkin, Ersin Çelik, Köksal Dönmez, Tayfun Göktoğan, Nagihan Karahan

**Affiliations:** 1 Department of Cardiovascular Surgery, Katip Celebi University Izmir Ataturk Training and Research Hospital, Izmir, Turkey

## Background/Introduction

Infective endocarditis, which is described as malignant disease of infections, has several complications increasing morbidity and mortality like splenic infarct and cerebral abscess.

## Aims/Objectives

Our case was 46 year-old male patient. Five before his admission to our clinic, he was hospitalized for weakness, headache, nausea and vomiting in a different clinic.

## Method

Transthoracic echocardiography revealed a vegetative lesion on mitral valve. Combined parenteralantiobiotheraphy was admitted. Blood cultures and antibiograms resulted with metisiline sensitive Staphylococcus Aureus and Pseudomonas Putida 10 days after. Patient was consulted to infectious disease department and continuation of antibiotics was decided. His abdominal CT revealed splenic infarct and cerebral CT revealed a 2 cm lesion located in left temporal lobe with peripheral edema. Cerebral MR examination revealed hypo intense cortical changes with hemorrhagic content that is settled by embolization. This was reported as an infective disease like cerebritis or multiple abscess formation. Neurosurgery consultation resulted as operation indication was going to be decided by patients response to antibiotheraphy and treatment of cardiac pathology has priority. In addition, they reported that elective surgery should planned after control cranial MR examination and there was not any risk for anticoagulation. Coronary angiography was normal but catheterization revealed serious mitral valve insufficiency.

## Results

Patient underwent surgery. After left atriotomy, there was a chorda rupture at P3 segment of posterior leaflet and vegetative lesion on anterior leaflet. Both of these findings were consistent with transeusophagial echocardiography. Native valve was excised. Due to its resistance to infection, short-term anticoagulation requirement, further operations, and advancements at valve in valve technique, bioprosthetic valve replacement was planned. By using 29 no St. Jude bioprothesis, valve was replaced by ti-cron sutures with pledgets. Patient recovered uneventfully.

## Discussion/Conclusion

We recommend multidisciplinary approach to infective endocarditis because of cardiac involvement, different complications types, patient specific problems, and pathogen related problems. Only by this method, optimal survival rates and complete eradication of pathogens may be achieved.

## Consent

Written informed consent was obtained from the patient for publication of this Case report and any accompanying images. A copy of the written consent is available for review by the Editor-in-Chief of this journal

